# Association between modified youth healthy eating index and nutritional status among Iranian children in Zabol city: a cross-sectional study

**DOI:** 10.1038/s41598-024-63038-3

**Published:** 2024-05-25

**Authors:** Farshad Amirkhizi, Mohammad-Reza Jowshan, Soudabeh Hamedi-Shahraki, Somayyeh Asghari

**Affiliations:** 1 Department of Nutrition, Faculty of Public Health, Zabol University of Medical Sciences, Zabol, Iran; 2 Department of Clinical Nutrition, School of Nutritional Sciences and Dietetics, Tehran University of Medical Sciences, No#44, Hojjatdoust St., Naderi St., Keshavarz Blvd, Tehran, 141556117 Iran; 3 Department of Epidemiology and Biostatistics, Faculty of Public Health, Zabol University of Medical Sciences, Zabol, Iran

**Keywords:** Healthy eating index, Nutrition status, Children, Underweight, Stunting, Wasting, Nutrition disorders, Nutrition, Public health

## Abstract

Diet quality in childhood and adolescence can affect health outcomes such as physical and cognitive growth and preventing chronic diseases in adulthood. This study aimed to evaluate the relationship between diet quality using the modified youth healthy eating index (MYHEI) with socioeconomic factors and nutrition status in 7–12-year-old children in Iran. This descriptive-cross-sectional study was performed on 580 students in Zabol, Iran, selected through multistage cluster sampling. The diet was assessed through the 168-item food frequency questionnaire (FFQ) and eating behaviors. Then, the MYHEI scoring system was used to calculate the diet quality. In addition, we used the WHO growth indices, such as weight to age, height to age, and body mass index (BMI) to age ratios, to evaluate nutrition status. The mean total MYHEI score in children was 56.3 ± 11.2. Among children with the highest MYHEI score quartile, the number of girls was significantly higher than boys (*p* = 0.001). The prevalence of underweight, stunting, and wasting was 25.3%, 17.4%, and 21.7%, respectively. The prevalence of underweight (OR: 2.2; 95% CI  1.26, 3.90, *p* = 0.001) and stunting (OR: 3.2; 95% CI   1.65, 6.14, *p* = 0.006) were significantly lower in the higher MYHEI score quartile compared to the lower quartile. The overall diet quality of most children should be modified. Therefore, to improve the children’s health and nutrition status, it is necessary to perform nutritional interventions such as training and promotional programs, especially in schools.

## Introduction

The emerging evidence in the last few decades has shown important changes in diet due to economic changes, urbanization, and globalization^1^. Based on the nutrition global reports in 2018, one out of three people is struggling with malnutrition, one out of 20 children is dealing with hunger and one out of five deaths is assigned to low diet quality^2^. These alterations have led to a double burden of malnutrition and an increased risk of chronic and non-communicable diseases including obesity, diabetes type 2, cardiovascular disease (CVD), and some types of cancer especially in low- and middle-income countries^3,4^. In Iran, the statistics of the Second National Integrated Micronutrients Survey (NIMS-II) in 2018 reported the prevalence of wasting and stunting as 8% and 5% respectively, and the prevalence of obesity or overweight was reported as approximately 16%^5^. In Zabol, underweight, wasting, and stunting among kindergarten were reported at 5%, 7.1%, and 7.3% respectively^6^.

Nutrition is one of the major influential factors in the development of cognitive, emotional, mental, and physical growth in childhood and early adolescence^7^. This part of life is the best opportunity to improve the nutrition status and prevent the health consequences caused by malnutrition and chronic diseases^8^. A dietary pattern institutionalized in this stage can continue until adulthood. That is why identifying different aspects of nutrition and the quality of the diet and its association with socio-economic and demographic factors is vital^9^. Several studies have indicated that a low-quality diet could affect mental and physical health as well as human and economic resources^10,11^. Since foods and macro- /micronutrients are not commonly consumed alone, investigating the dietary patterns makes it possible to study the impact of the whole diet on nutritional status which provides a practical approach for dietary recommendations to improve overall health^12^.

The Healthy Eating Index (HEI) has been considered to assess the diet quality in different societies with different food patterns. On the other hand, the Modified Youth HEI (MYHEI) scoring system was developed to rate the diet quality in the population of children and adolescents^13^ which was modified and validated for the Iranian children and adolescents by Mirmiran et al^14^. Higher MYHEI scores indicate the consumption of nutrient-dense and healthy foods. Given the impact of nutrition status on health outcomes and shaping nutrition habits during childhood, evaluation of the diet quality is of high importance. On the other hand, studies evaluating the association of MYHEI scores with nutritional status among Iranian children and adolescents are limited. So, the current study was performed to evaluate the relationship between the quality of the diet using MYHEI with some socio-demographic factors and nutritional outcomes including stunting, wasting, and being underweight as well as energy and nutrient intakes among 7–12-year-old Iranian children.

## Materials and methods

### Study subjects

In the present cross-sectional study, 580 school children aged 7–12 years were recruited from May to September 2021 by a multi-stage cluster sampling method as a representative sample of children in the urban areas of Zabol, Sistan and Baluchestan Province in the southeast of Iran. At first, schools were selected as clusters, and then school classes were considered as strata. Afterward, the children were randomly chosen from the list of student records of each class.

The sample size of the study was calculated based on information obtained from the study by Shahraki et al^15^. By considering a confidence interval of 95% (*z*), a degree of precision of 3% (*d*), and a prevalence of stunting of 16% (*p*), it was calculated that 574 subjects needed to be included in the study. Estimating a nonresponse rate of 5%, 602 children were invited to participate in this study. The inclusion criteria were being in the age range of 7 to 12 years and the willingness of child parents to participate in the study. The exclusion criteria included taking any medications, being on a specific diet, and having any hereditary disorders or chronic diseases. After excluding those with implausible energy intakes of lower than 500 kcal/day and higher than 4000 kcal/day (12 boys, 10 girls), 580 children remained for the analyses.

### Demographic data and anthropometric measurements

General characteristics of children and their parents including age and sex, parent’s educational level, parent’s occupation, household size, and number of children in the household were obtained through face-to-face interviews with the child’s mother by two trained research assistants using a questionnaire.

The weight of participants was measured without shoes in light clothing using a Beurer digital weighing scale (Beurer BF66, Germany). Also, height was measured in a standing position without shoes by mounting tape to the nearest 0.1 cm precision. Body mass index (BMI) was calculated as weight (kg) divided by squared height (m^2^). The weighing scale was calibrated to zero before taking every measurement. To minimize the subjective error, all measurements were done by the same person.

### Assessment of dietary intake

Dietary intake was evaluated using a semi-quantitative food frequency questionnaire (FFQ) with 168 food items, which was designed and validated specifically for the Iranian population^14^. The good validity and reliability of the FFQ in terms of assessing the nutrient and food consumption of Iranian children has been reported previously^16^. The FFQ also evaluated eating behaviors relevant to the diet quality of children, including the use of sugar-sweetened beverages, sweet snacks, salty snacks, and the frequency of eating fast foods.

The trained research assistants administered all the questionnaires by interviewing the children and their mothers. The mothers were asked about the consumption of a given serving of each food item by children during the previous year on a daily (*e.g.* bread), weekly (*e.g.* rice, milk), or monthly (*e.g.* fish) basis. The research assistants guided the child’s mothers on the estimation of food quantities, using a set of calibrated household measurements (*e.g.* cups, glasses, bowls, plates, spoons, ladles). Portion sizes of consumed foods were then converted to the gram, using household measures. Food intakes were then converted to energy and other nutrients using the Nutritionist-IV (N4) software program (version 7.0; N-Squared Computing, Salem, OR, USA)^17,18^, modified for Iranian foods. Almost all foods eaten by the subjects could be coded. When a particular ethnic food was not in the database of N4, it was coded as a similar item.

### Assessment of adherence to the modified youth healthy eating index

Adherence of the participants to the healthy eating guidelines was assessed using the MYHEI scoring system. Briefly, MYHEI consisted of 10 components including whole grains, fruits, vegetables, dairy, meat ratio, sugar-sweetened beverages, butter and margarine, sweet snacks, salty snacks, and fast foods. In the MYHEI scoring system, higher consumption of the five components (whole grains, fruits, vegetables, dairy, and meat ratio) and avoidance or lower consumption of the remaining five components (sugar-sweetened beverages, butter and margarine, sweet snacks, salty snacks, and fast foods) indicate a healthier diet. Each component is scored 0 (for lack of adherence) to 10 (for full adherence), with intermediate scores calculated to indicate the degree of adherence to dietary recommendations. All the component scores are summed to obtain a total MYHEI score, which ranges from 0 to 100, with a higher score indicating a healthier diet. The criteria for scoring each component are summarized in Table 1.

**Table 1:** Components and scoring criteria of Modified Youth Healthy Eating Index.

MYHEI component	Maximum points	Requirements for maximum score of 10	Requirements for minimum score of zero
Servings/day			
Whole grains^a^	10	≥ 2	0
Fruits^b^	10	≥ 3	0
Vegetables^c^	10	≥ 3	0
Dairy^d^	10	≥ 3	0
Meat ratio^e^	10	≥ 2	0
Sugar sweetened beverages^f^	10	0	≥ 2
Grams/day			
Butter and margarine	10	0	≥ 10
Sweet snacks^g^	10	≤ 8.4	≥ 111.9
Salty snacks^h^	10	≤ 1.6	≥ 50.7
Fast foods^i^	10	≤ 1.4	≥ 25.0
MYHEI total (0–100)			

^a^Includes dark breads (sangak, barbari, taftoon), barley bread, cornflakes, bulgur and germs.

^b^ Includes apples, oranges, bananas, peaches, grapes, strawberries, pears, watermelon, grapefruit, prunes, kiwi, figs, pomegranates, persimmons, berries, raisins, coconuts, apricots and sweet lemons.

^c^ Includes onions, cucumbers, lettuces, carrots, cauliflower, Brussels sprouts, kale, cabbage, spinach, mixed vegetables, corn, green beans, green peas, peppers, beets, potatoes, tomatoes, broccoli and celery.

^d^ Includes milk, yoghurt and cheeses.

^e^Total number of servings per day of chicken, fish, eggs, nuts, seeds, soy, and beans divided by the total number of servings/day of beef, Mutton, lamb, hearts, kidneys, and liver.

^f^ Includes any beverages with added sugar (*e.g*. artificially sweetened beverages and sugar sweetened fruit juice).

^g^Includes snacks with added sugar (*e.g*. cake, snack cake, toaster pastry, sweet roll/danish/pastry, doughnut, cookies, pie, chocolate, candy bar with chocolate, candy without chocolate, fruit rollup, popsicle, and flavored gelatin).

^h^ Includes salty snacks (*e.g*. potato chips, corn chips, nachos, popcorn, pretzels, and crackers).

^i^Includes sausages, cold cuts, hamburgers, pizza, fried chicken, french fries, fried and gravy mushroom, onion rings, chicken nuggets, hot dogs, chicken ham.

### Assessment of nutritional outcomes

In this study, underweight, stunting, and wasting as nutritional outcomes were investigated in the participants. Nutritional outcomes of children were evaluated by calculating weight-for-age (WAZ), height-for-age (HAZ), and BMI-for-age (BAZ) Z scores according to the World Health Organization (WHO) growth standards 2007 for 5–19 years (WHO 2007). The Z scores for these nutritional indicators were calculated using the WHO Anthro Plus software program (version 1.0.4)^19^. Underweight, stunting, and wasting among children were considered as WAZ, HAZ, and BAZ less than 2 standard deviations (Z score < -2SD) below the median of the reference population (growth references, WHO 2007), respectively^19^.

### Statistical analysis

Data were analyzed using IBM SPSS version 25 (IBM Corp., Armonk, NY, USA). Participants were classified based on cut-points of MYHEI in quartiles categories as follows: 1st, < 47.5; 2nd, 47.5 to < 54.5; 3rd, 54.5 to < 64.4; 4th, ≥ 64.4. The Kolmogorov–Smirnov test was applied to determine the normality of the data. The results are presented as mean ± standard deviation for quantitative data with normal distribution; while for qualitative data, frequency (percent) was used. Noticeable variations in anthropometric and general characteristics across quartile groups of MYHEI were assessed by the ANOVA with Tukey’s post hoc test. To identify considerable differences, the Pearson chi-square (χ^2^) test was employed across quartile groups of MYHEI for qualitative data.

Age-, sex- and energy-adjusted intakes of nutrients and food groups were compared across the MYHEI categories using analysis of covariance (ANCOVA), with Bonferroni correction. The Kruskal–Wallis test was used to compare numerical variables with non-normal distribution. The association of MYHEI component scores and nutritional outcomes among participants were also investigated by ANCOVA, after adjusting for age, sex, and energy intake. To explore the relationship between MYHEI and the probability of underweight, stunting, and wasting, multivariate logistic regression was used in crude and adjusted models. In the adjusted model, important factors including age (years), sex (boy/girl), and energy intake (kcal/day) were controlled. In all multivariate models, the fourth quartile (Q_4_) of the MYHEI was considered as a reference. The Mantel–Haenszel extension test was used to evaluate the overall trend of increasing quartile categories of MYHEI associated with an increasing prevalence of nutritional outcomes. The findings of logistic regression are described as adjusted odds ratios (ORs) with 95% confidence intervals (CIs). A *p*-value < 0.05 was defined as significant.

### Ethics approval and consent to participate

All the methods and procedures carried out in this study were by the guidelines of the Declaration of Helsinki and approved by the Ethics Committee of Zabol University of Medical Sciences (Ethics code: IR.ZBMU.REC.1401.006). Before the beginning of the study, children’s parents were fully informed about the objectives and protocol of the study. Parents and guardians provided informed written consent for their children participating in the study. Parents were informed that they could revoke the participation agreement at any time.

## Results

The study sample consisted of 580 children with a mean (± standard deviation) of 9.6 ± 1.6 years. Of this children population, 48.1% (*n* = 279) were boys and 51.9% (*n* = 301) were girls. The general characteristics of children and their households across quartiles of MYHEI scores are presented in Table 2. Children in the highest quartile of MYHEI were more likely to be girls (*p* = 0.001) and had significantly higher weight, height, and BMI (*p* < 0.0001 for all) compared with those in the lowest quartile. Furthermore, children in the second and third quartiles of MYHEI had higher weight and BMI compared with those in the first quartile (*p* < 0.05 for all). There were no significant differences regarding education levels and occupation status of children’s parents, household size, and the number of children per household across categories of MYHEI.

**Table 2:** General characteristics of children and their households across quartiles (Q) of Modified Youth Healthy Eating Index scores^a^.

Variables	Overall (*n* = 580)	Quartile categories of MYHEI^b^				*p*^c^
		Q1 (*n* = 146) (Unhealthiest)	Q2 (*n* = 145)	Q3 (*n* = 144)	Q4 (*n* = 145) (Healthiest)	
Sex of the children, *n* (%)						
Boy	279 (48.1)	87 (59.6)	70 (48.3)	62 (43.1)	60 (41.4)	0.001
Girl	301 (51.9)	59(40.4)	75 (51.7)	82 (56.9)	85 (58.6)	
Age of the children (years)	9.6 ± 1.6	9.3 ± 1.7	9.7 ± 1.6	9.4 ± 1.5	9.9 ± 1.6	0.088
Weight (kg)	29.7 ± 7.7	27.0 ± 6.5	29.7 ± 7.6*	29.9 ± 7.5*	32.0 ± 8.4**	< 0.0001
Height (cm)	132 ± 10	129 ± 9	131 ± 10	133 ± 9*	135 ± 10**	< 0.0001
BMI (kg/m^2^)	16.7 ± 2.6	15.9 ± 2.2	16.9 ± 2.6*	16.7 ± 2.4*	17.2 ± 2.8**	< 0.0001
Education level of mother, *n* (%)						
No schooling	31 (5.3)	7 (4.8)	10 (6.9)	3 (2.1)	11 (7.6)	0.755
Primary	204 (35.2)	65 (44.5)	38 (26.2)	44 (30.6)	57 (39.3)	
Secondary	230 (39.7)	40 (27.4)	71 (49.0)	68 (47.2)	51 (35.2)	
Diploma& university	115 (19.8)	34 (23.3)	26 (17.9)	29 (20.1)	26 (17.9)	
Education level of father, *n* (%)						
No schooling	30 (5.2)	10 (6.8)	8 (5.5)	5 (3.5)	7 (4.8)	0.280
Primary school	132 (22.8)	41 (28.1)	30 (20.7)	26 (18.0)	35 (24.2)	
Secondary & high school	227 (39.1)	52 (35.6)	55 (37.9)	62 (43.1)	58 (40.0)	
Diploma& university	191 (32.9)	43 (29.5)	52 (35.9)	51 (35.4)	45 (31.0)	
Mother’s occupation status, *n* (%)						
Housewife	472 (81.4)	118 (80.8)	113 (77.9)	117 (81.3)	124 (85.5)	0.229
Working	108 (18.6)	28 (19.2)	32 (22.1)	27 (18.8)	21 (14.5)	
Father ‘s occupation status, *n* (%)						
Jobless	33 (5.7)	12 (8.2)	9 (6.2)	5 (3.5)	7 (4.8)	0.464
Worker/ farmer	137 (23.6)	40 (27.4)	27 (18.6)	36 (25.0)	34 (23.4)	
Employee	158 (27.2)	32 (21.9)	45 (31.0)	34 (23.6)	47 (32.4)	
Self-employed	252 (43.4)	62 (42.5)	64 (44.1)	69 (47.9)	57 (39.3)	
Household size	5.9 ± 1.5	6.0 ± 1.7	5.9 ± 1.5	5.7 ± 1.5	5.8 ± 1.5	0.205
No. of children per household	3.7 ± 1.5	3.8 ± 1.7	3.7 ± 1.5	3.6 ± 1.4	3.7 ± 1.5	0.449

MYHEI, Modified Youth Healthy Eating Index.

^a^Values are presented as mean and standard deviation unless indicated otherwise.

^b^Quartile cut-points of MYHEI are as follows: 1st quartile, < 47.5; 2nd quartile, 47.5 to < 54.5; 3rd quartile, 54.5 to < 64.4; 4th quartile, ≥ 64.4

^c^Obtained from ANOVA for continuous variables and χ^2^ test for categorical variables (*P* < 0.05 was considered significant).

*P < 0.05 compared with 1st quartile (post hoc Tuky’s test).

**P < 0.0001 compared with 1st quartile (post hoc Tuky’s test).

Age, sex, and energy-adjusted means for selected nutrients and food groups across quartiles of MYHEI are shown in Table 3. Children in the highest quartile of MYHEI compared with the lowest quartile had significantly higher intakes of energy (*p* < 0.0001), protein (*p* < 0.05), cholesterol (*p* < 0.05), vitamin A (*p* < 0.0001), vitamin B_6_ (*p* < 0.05), folate (*p* < 0.0001), calcium (*p* < 0.0001), and zinc (*p* < 0.0001). Likewise, children in the third quartile of MYHEI had higher intakes of energy, protein, vitamin A, vitamin B_6_, folate, calcium, and zinc compared with those in the first quartile (*p* < 0.05 for all). Greater adherence to MYHEI was associated with higher consumption of fruits and dairy products. Those in the third or fourth quartiles of MYHEI had significantly higher consumption of fruits (*p* < 0.0001) and dairy products (*p* < 0.05) compared with those in the first quartile. There was no significant difference regarding intakes of carbohydrates, total fat, SFA, vitamin C, iron, magnesium, and consumption of meats, vegetables, nuts, and legumes across MYHEI quartiles (Table 3).

**Table 3:** Energy and nutrient intakes of children across quartiles (Q) of Modified Youth Healthy Eating Index scores^a^.

Nutrients	Overall (*n* = 580)	Quartile categories of MYHEI^b^				*p*^c^
		Q1 (*n* = 146) (Unhealthiest)	Q2 (*n* = 145)	Q3 (*n* = 144)	Q4 (*n* = 145) (Healthiest)	
Energy intake (kcal/d)^d^	1565 ± 154	1502 ± 158	1542 ± 141	1594 ± 153*	1627 ± 165**^,^***	< 0.0001
Nutrients						
Protein (g/d)	43.1 ± 5.6	34.3 ± 6.7	41.5 ± 5.7	48.2 ± 5.1*	50.5 ± 5.6*	0.006
Carbohydrate (g/d)	252 ± 45	241 ± 46	246 ± 40	259 ± 47	263 ± 45	0.092
Fat (g/d)	49.3 ± 10.1	46.3 ± 10.8	48.4 ± 9.5	50.8 ± 10.2	49.9 ± 9.5	0.151
SFA (g/d)	21.3 ± 7.8	21.9 ± 7.5	20.3 ± 7.6	19.8 ± 8.1	22.5 ± 7.8	0.347
Cholesterol (g/d)	132 ± 89	127 ± 86	134 ± 85	133 ± 90	140 ± 95*	0.016
Vitamin A (µg/d)	480 (362–825)	439 (316–756)	480 (368–812)	497 (355–824)*	506 (406–890)**	< 0.0001
Vitamin C (mg/d)	42.5 ± 12.2	39.5 ± 12.9	42.7 ± 15.6	45.1 ± 10.2	40.8 ± 11.3	0.406
Vitamin B_6_ (mg/d)	1.13 (0.88–1.87)	0.83 (0.73–1.37)	1.05 (0.84–1.87)	1.27 (0.90–1.97)*	1.38 (1.06–2.18)*	0.014
Folate (µg/d)	217 (167–372)	197 (131–298)	214 (169–361)	234 (194–407)*	250 (203–421)**^,^***	< 0.0001
Iron (mg/d)	11.4 ± 3.5	10.6 ± 2.5	11.2 ± 2.4	12.5 ± 2.1	11.5 ± 7.8	0.125
Calcium (mg/d)	1140 ± 584	1074 ± 614	1107 ± 608	1135 ± 616*	1256 ± 501**^,^***	< 0.0001
Zinc (mg/d)	8.1 ± 2.6	6.8 ± 2.6	7.5 ± 2.8	8.8 ± 2.3*	9.5 ± 2.8**^,^***	< 0.0001
Magnesium (mg/d)	252 ± 62	246 ± 56	251 ± 60	249 ± 67	264 ± 64	0.109
Food groups						
Meats (g/d)	86.5 ± 3.4	83.2 ± 2.5	88.6 ± 3.2	84.8 ± 4.1	90.5 ± 3.8	0.129
Fruits (g/d)	124 ± 71	109 ± 72	119 ± 70	130 ± 68**	134 ± 70**^,^***	< 0.0001
Vegetables (g/d)	194 ± 67	181 ± 64	193 ± 69	204 ± 71	198 ± 65	0.263
Nuts and legumes (g/d)	55.2 (44.8–77.9)	55.8 (43.7–79.3)	49.5 (38.4–70.6)	57.4 (45.9–79.6)	60.3 (51.0–82.1)	0.175
Dairy products (g/d)	187 ± 49	179 ± 49	185 ± 51	191 ± 47*	203 ± 50*	0.005

MYHEI, Modified Youth Healthy Eating Index; SFA, saturated fatty acid.

^a^Values are presented as mean ± standard deviation for normally distributed data and median (IQR) for data not normally distributed (*i.e.* vitamin A, vitamin B_6_, folate, nuts and legumes).

^b^Quartile cut-points of MYHEI are as follows: 1st quartile, < 47.5; 2nd quartile, 47.5 to < 54.5; 3rd quartile, 54.5 to < 64.4; 4th quartile, ≥ 64.4

^c^Obtained from ANCOVA for normally distributed data and Kruskal–Wallis test for data not normally distributed (*i.e.* vitamin A, vitamin B6, folate, nuts and legumes).

^d^Energy intake is adjusted for sex and age; all other values are adjusted for age, sex and energy intake.

*P < 0.05 compared with 1st quartile (post hoc Sidac test).

**P < 0.0001 compared with 1st quartile (post hoc Sidac test).

***P < 0.05 compared with 2nd quartile (post hoc Sidac test).

**Figure 1:**
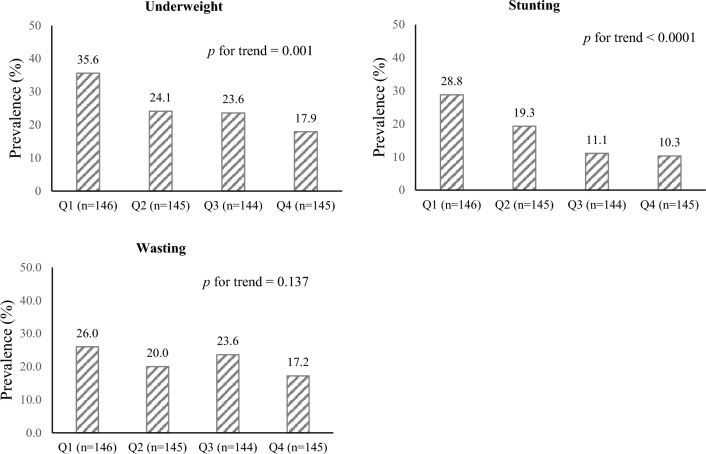
The prevalence of underweight, stunting, and wasting among children across the quartiles of Modified Youth Healthy Eating Index scores (MYHEI). Quartile cut-points of MYHEI (Q) are as follows: 1st quartile, < 47.5; 2nd quartile, 47.5 to < 54.5; 3rd quartile, 54.5 to < 64.4; 4th quartile, ≥ 64.4. *P*-values derived from Mantel-Hanszel extension χ^2^ test.

The prevalence of underweight, stunting, and wasting among participants was 25.3%, 17.4%, and 21.7%, respectively. There was no significant difference regarding the prevalence of the aforementioned nutritional outcomes between girls and boys (data not shown). The prevalence of underweight, stunting, and wasting across quartiles of MYHEI is shown in Fig. 1, As can be seen, underweight (*p*-trend = 0.001) and stunting (*p*-trend < 0.0001) were significantly lower prevalent among participants in the highest quartile of MYHEI compared with those in the bottom quartile. Indeed, greater adherence to MYHEI was associated with a lower prevalence of underweight and stunting in the children. No significant associations were found between adherence to MYHEI and the prevalence of wasting among the participants.

The association of MYHEI component scores and nutritional outcomes among children is presented in Table 4. The mean (± standard deviation) of the MYHEI total score was 56.3 ± 11.2. The highest and lowest scores of MYHEI components were related to “whole grains” (8.3 ± 1.3) and “meat ratio” (2.7 ± 1.6), respectively. The mean scores of “whole grains” (*p* < 0.0001), “meat ratio” (*p* = 0.008), and “sweet snacks” (*p* = 0.034) were significantly lower in underweight children compared with normal-weight children, after adjustment for confounders. Compared to the children with normal height growth, those who were stunted had significantly lower scores for “fruits” (*p* < 0.0001), “dairy” (*p* = 0.001), “meat ratio” (*p* = 0.005) and higher scores for “butter and margarine” (*p* = 0.001). No significant differences were found between wasted and normal children regarding the scores of MYHEI components. After adjustment for confounding factors, MYHEI’s total score in both underweight (*p* = 0.001) and stunted (*p* < 0.0001) children were significantly lower than children with normal growth. There were no significant associations between MYHEI total score and wasting among participants.

**Table 4:** Association of modified youth healthy eating index component scores and nutritional outcomes among children^a^.

MYHEI components	Overall (n = 580)	Nutritional outcomes								*p*^c^
		Underweight^b^ (n = 147)	Normal (n = 433)	*p*^c^	Stunting^b^ (n = 101)	Normal (n = 479)	*p*^c^	Wasting^b^ (n = 126)	Normal (n = 454)	
Serving/day										
Whole grains	8.3 ± 1.3	7.5 ± 1.4	8.4 ± 1.3	< 0.0001	7.9 ± 1.5	8.6 ± 1.3	0.093	8.0 ± 1.4	8.7 ± 1.4	0.184
Fruits	5.3 ± 1.7	5.2 ± 1.7	5.5 ± 1.7	0.101	4.7 ± 1.7	5.5 ± 1.6	< 0.0001	5.0 ± 1.7	5.4 ± 1.7	0.135
Vegetables	6.2 ± 1.7	5.9 ± 1.7	6.3 ± 1.7	0.091	5.9 ± 1.6	6.5 ± 1.6	0.203	6.0 ± 1.7	6.4 ± 1.7	0.075
Dairy	5.5 ± 1.7	5.3 ± 1.6	5.7 ± 1.9	0.121	5.0 ± 1.5	5.6 ± 1.8	0.001	5.3 ± 1.8	5.6 ± 1.7	0.260
Meat ratio	2.7 ± 1.6	2.4 ± 1.5	3.1 ± 1.6	0.008	2.3 ± 1.4	3.2 ± 1.6	0.005	2.6 ± 1.5	2.8 ± 1.6	0.151
Sugar sweetened beverages	7.5 ± 1.5	7.4 ± 1.6	7.5 ± 1.5	0.532	7.1 ± 1.5	7.5 ± 1.6	0.101	7.4 ± 1.6	7.5 ± 1.5	0.373
Grams/day										
Butter and margarine	5.9 ± 1.6	5.7 ± 1.6	6.2 ± 1.7	0.290	6.4 ± 1.4	5.2 ± 1.7	0.001	5.4 ± 1.7	6.5 ± 1.6	0.002
Sweet snacks	5.0 ± 1.4	4.8 ± 1.3	5.3 ± 1.4	0.034	5.3 ± 1.3	4.7 ± 1.4	0.101	4.8 ± 1.3	5.1 ± 1.4	0.092
Salty snacks	5.0 ± 1.5	4.9 ± 1.6	5.1 ± 1.5	0.070	5.1 ± 1.4	4.6 ± 1.5	0.140	4.9 ± 1.4	5.1 ± 1.5	0.255
Fast foods	4.7 ± 1.6	4.4 ± 1.9	4.8 ± 1.9	0.082	4.8 ± 2.0	4.1 ± 1.9	0.125	4.4 ± 1.8	4.8 ± 2.0	0.069
MYHEI total	56.3 ± 11.2	53.8 ± 10.6	57.2 ± 11.2	0.001	50.5 ± 10.0	57.5 ± 11.1	< 0.0001	54.6 ± 10.6	56.8 ± 11.3	0.055

MYHEI, Modified Youth Healthy Eating Index.

^a^Values are presented as mean ± standard deviation.

^b^ Z-score < -2SD from the median of WHO (2007) growth reference (5–19 years).

^c^Obtained from ANCOVA after controlling for age, sex and energy intake (*p* < 0.05 was considered significant).

Table 5 indicates the odds ratio (OR) and 95% confidence interval (CI) for the incidence of underweight, stunting, and wasting as the dependent variables across quartiles of MYHEI as independent variables. In the crude model, the probability of having underweight (OR, Q_1_-Q_4_: 2.5, 1.5, 1.4, 1.0, respectively; *p*-trend = 0.001) and stunting (OR, Q_1_-Q_4_: 3.5, 2.1, 1.2, 1.0, respectively; *p*-trend < 0.0001) elevated with decreasing quartile of the MYHEI. The chance of having underweight (OR, Q_1_-Q_4_: 2.2, 1.4, 1.3, 1.0, respectively; *p*-trend = 006) and stunting (OR, Q_1_-Q_4_: 3.2, 2.0, 1.1, 1.0, respectively; *p* = 0.001) significantly increased even after adjustment for age, sex and energy intake.

**Table 5:** Crude and adjusted OR (95%CI) for incidence of underweight, stunting and wasting among children across quartiles (Q) of Modified Youth Healthy Eating Index scores.

Nutritional outcomes and MYHEI-score	Simple logistic regression	Multiple logistic regression
	Crude OR (95.0% C.I.)	Adj. OR (95.0% C.I.)^b^
Underweight^c^		
Q1^d^	2.5 (1.47, 4.36)	2.2 (1.26, 3.90)
Q2	1.5 (0.82, 2.57)	1.4 (0.77, 2.48)
Q3	1.4 (0.80, 2.51)	1.3 (0.74, 2.49)
Q4 (Reference)	1.0	1.0
* P*-trend^a^	0.001	0.006
Stunting^c^		
Q1	3.5 (1.84, 6.66)	3.2 (1.65, 6.14)
Q2	2.1 (1.10, 4.07)	2.0 (1.02, 3.96)
Q3	1.2 (0.51, 2.28)	1.1 (0.50, 2.24)
Q4 (Reference)	1.0	1.0
* P*-trend^a^	< 0.0001	0.001
Wasting^c^		
Q1	1.7 (0.96, 2.98)	1.4 (0.77, 2.47)
Q2	1.2 (0.66, 2.17)	1.1 (0.61, 2.03)
Q3	1.5 (0.83, 2.64)	1.4 (0.79, 2.56)
Q4 (Reference)	1.0	1.0
* P*-trend^a^	0.137	0.282

OR, odds ratio; CI, confidence interval.

^a^*p* < 0.05 was considered significant.

^b^Adjusted for age, sex, and energy intake.

^c^ Z-score < -2SD from the median of WHO (2007) growth reference (5–19 years).

^d^ Quartile cut-points of MYHEI are as follows: 1st quartile, < 47.5; 2nd quartile, 47.5 to < 54.5; 3rd quartile, 54.5 to < 64.4; 4th quartile, ≥ 64.4

## Discussion

The current study investigated the connection between MYHEI and nutritional status and social-demographic factors in 7–12-year-old Iranian children. Results showed that the prevalence of underweight, stunting, and wasting among participants was 25.3%, 17.4%, and 21.7%, respectively, with no differences between girls and boys. Based on the findings, children in the highest quartile of MYHEI had significantly higher weight, height, and BMI with a lower prevalence of underweight and stunting. To the best of our knowledge, limited studies are available investigating the association between MYHEI and nutritional status in Iranian children.

Similar to this study findings, Koksal et al. determined a positive association between MYHEI score and weight, height, and BMI^20^. However, Askari et al. could not find any relationship between HEI scores and these anthropometric measurements in a cross-sectional study of 788 six-year-old children^11^ which was also observed in several other studies that used HEI^21–24^. It seems that MYHEI is a better predictor for nutritional status than older HEI versions since salty snacks and sweet beverages and snacks, the core elements of MYHEI, are more frequently consumed by children, which may result in a lack of enough micronutrient intake and a higher rate of stunting^25^. Children in the highest quartile of MYHEI had significantly higher intakes of energy, protein, cholesterol, vitamin A, vitamin B_6_, folate, calcium, and zinc.

Furthermore, underweight children had significantly lower scores for “whole grains”, “meat ratio”, and “sweet snacks” and stunted children had lower scores for “fruits”, “dairy”, and “meat ratio” as well as a higher score for “butter and margarine” compared with normal children. Lower cereal consumption reduces macro- and micro-nutrient intakes including carbohydrate, protein, magnesium, vitamin E, calcium, and potassium which could result in the subjects being underweight. This implies adequate cereal intake might be essential for a weight balance in children^26–28^. On the other hand, it seems that inadequate intake of macronutrients and micronutrients may cause stunting, so the weight-to-height index (wasting) does not change with the lower intake of nutrients, while it may cause underweight, as indicated in current study findings.

MYHEI interestingly does not measure energy intake. However, this score is highly related to the intake of several essential nutrients and proteins necessary for the growth and health of children, which shows that higher MYHEI scores signify a better diet quality alongside its relation and impact on health and nutrition status^9,29,30^. Furthermore, participants in the highest MYHEI quartile reported considerably higher fruit and dairy intakes than the lower MYHEI quartile. These higher dairy intakes are rather important in teenagers due to being in their growth stage. These findings match some other studies^11,31^.

In accordance with the present study results, some of the previous studies also reported that females usually achieve higher MYHEI scores^32–34^. It has been shown that boys are less inclined to eat healthy foods to control their body weight. However, some studies did not support this conclusion^31,35–37^. No significant differences were found between household size, the number of children per household, and education levels and occupation status of children’s parents with MYHEI. Furthermore, unlike other studies, there was no other meaningful relationship between demographic and economic features such as education level, parent job status, family size, and the number of children in each family of MYHEI groups^7,38–40^.

Manios et al. in a study conducted on the Greek population, observed lower HEI scores in unemployed or less educated (less than 9 years) mothers compared to educated mothers (more than 9 years) in 2518 1–5-year-old children^7^. On the other hand, in a study on 326 students aged 13–17 years old by Yaghoobloo et al., there was no correlation between the parent's education level and their children's diet quality^31^. This lack of correlation could be due to the social conditions of adolescents to choose foods without adhering to parental guidance or the lack of connection between educational status and income in developing countries. Furthermore, this could reflect the cross-sectional study type that cannot observe and conclude the causality relationship between the MYHEI score and parental education.

This study has a variety of strengths and limitations. It should be noted that studies using MYHEI in analyzing diet quality in 7–12-year-old Iranian children are limited. Considering confounding factors in data analysis, proper sample size alongside the use of an authentic and reliable FFQ for diet analysis, trained interviewers, using standard methods in evaluation, as well as using the stricter MYHEI index for diet quality analysis compared to previous healthy diet indexes are the strengths of this study.

However, this is a cross-sectional study, which prevents the creation of any causal relationships. Furthermore, this study might have some common nutrition study biases, such as failure to report unhealthy food consumption properly, excessive healthy food reports, and lower or higher food consumption reports by parents or caretakers. This study uses multiple methods to facilitate diet reminders and reduce errors. Furthermore, not considering the effects of unknown confounding variables and data collection in a specific seasonal period might affect normal food intake. Also, some studies use 11–12-year-old students themselves with perception, conceptualization, memory skills, and accurate meal size estimation^41^. This might lead to more accurate reporting compared to parents.

## Conclusion

The current study suggested a meaningful relationship between MYHEI and gender, anthropometric parameters, alongside the lower prevalence of stunting and underweight in 7–12-year-old Iranian students. However, there was no relationship with other indexes such as socioeconomic status, family education levels, and wasting prevalence. These findings might help develop health promotions and nutritional interventions, especially in schools to improve this population's diet quality. Future studies could further investigate the relationship between the MYHEI quality index with nutritional outcomes and general characteristics in childhood and adolescence.

## Data Availability

On reasonable request, the corresponding author will provide the datasets used and analyzed during the current work.

## Abbreviations

MYHEI: Modified youth healthy eating index
MYHEI: Youth healthy eating index
HEI: Healthy eating index
FFQ: Food frequency questionnaire
BMI: Body mass index
CVD: Cardiovascular disease
NIMS-II: Second national integrated micronutrients survey
N4: Nutritionist-IV
WAZ: Weight-for-age
HAZ: Height-for-age
BAZ: BMI-for-age
WHO: World health organization
ANCOVA: Analysis of covariance
Q_4_: Quartile
ORs: Adjusted odds ratios
CIs: Confidence intervals
SFA: Saturated fatty acids
